# Development and Evaluation of the Brief Sexual Openness Scale—A Construal Level Theory Based Approach

**DOI:** 10.1371/journal.pone.0136683

**Published:** 2015-08-26

**Authors:** Xinguang Chen, Yan Wang, Fang Li, Jie Gong, Yaqiong Yan

**Affiliations:** 1 University of Florida, Gainesville, Florida, United States of America; 2 Wuhan Centers for Disease Prevention and Control, Wuhan, Hubei, China; Catholic University of Sacred Heart of Rome, ITALY

## Abstract

Obtaining reliable and valid data on sensitive questions represents a longstanding challenge for public health, particularly HIV research. To overcome the challenge, we assessed a construal level theory (CLT)-based novel method. The method was previously established and pilot-tested using the Brief Sexual Openness Scale (BSOS). This scale consists of five items assessing attitudes toward premarital sex, multiple sexual partners, homosexuality, extramarital sex, and commercial sex, all rated on a standard 5-point Likert scale. In addition to self-assessment, the participants were asked to assess rural residents, urban residents, and foreigners. The self-assessment plus the assessment of the three other groups were all used as subconstructs of one latent construct: sexual openness. The method was validated with data from 1,132 rural-to-urban migrants (mean age = 32.5, SD = 7.9; 49.6% female) recruited in China. Consistent with CLT, the Cronbach alpha of the BSOS as a conventional tool increased with social distance, from .81 for self-assessment to .97 for assessing foreigners. In addition to a satisfactory fit of the data to a one-factor model (CFI = .94, TLI = .93, RMSEA = .08), a common factor was separated from the four perspective factors (i.e., migrants’ self-perspective and their perspectives of rural residents, urban residents and foreigners) through a trifactor modeling analysis (CFI = .95, TLI = .94, RMSEA = .08). Relative to its conventional form, CTL-based BSOS was more reliable (alpha: .96 vs .81) and valid in predicting sexual desire, frequency of dating, age of first sex, multiple sexual partners and STD history. This novel technique can be used to assess sexual openness, and possibly other sensitive questions among Chinese domestic migrants.

## Introduction

### Challenges to assessing sexual risks in HIV research

Measuring sensitive questions is a significant challenge in public health research, particularly for HIV-related survey studies [1]. Most research questions regarding HIV risk are sensitive because they include topics such as disclosure of HIV status, social stigma, drug use history, sexual orientation, and sexual behavior. A large amount of data has been collected on many variables related to the risk of HIV infection, such as the number of sexual partners, type and frequency of sexual intercourse, engagement in commercial sex, extramarital sex, homosexual sexual encounters, alcohol and drug use prior or during sex, condom use, voluntary testing, and adherence to treatment. However, the reliability and validity of these data are often questionable due to their sensitive nature [2].

Evidence suggests that there is a positive relationship between permissive sexual attitudes and a number of sexual risk behaviors, including premarital sex, multiple sexual partners, and extramarital sex [3]. This relationship implies a measurement alternative: evaluating sexual attitudes rather than the detailed, explicitly described sexual acts because the former is more sensitive than the latter. In this study, we used sexual openness as an example to assess a novel approach we previously pilot-tested for survey studies to collect data on sensitive questions.

### Construal levels and sensitivity

Several methods have been attempted to better assess sensitive questions in research, such as the indirect questioning technique [4], the randomized response technique [5], the nominative technique [6], and the bogus pipeline procedure [7]. Application of these methods is limited; probably due to the complex procedures and/or ethical concerns [1]. Furthermore, most of these methods attempt to circumvent the sensitive nature of a survey question rather than directly tackle it to ensure reliability and validity.

In terms of the psychology of survey responses, a question’s sensitivity is conditioned on an individual’s perceived intrusiveness, threat of disclosure, and social desirability [1, 8], which is closely related to construal levels [9]. Construals are mental constructions of the universe at different psychosocial and physical distances with self, here and now as the reference point [10, 11] and construal level theory (CLT) suggests that a question’s sensitivity is not fixed, but rather varies negatively with social distance [10–12]. Relative to an assessment of oneself that occurs at the lowest construal level, a question would become less sensitive if it is used to assess socially distant others which occurs at higher construal levels—a process called *desensitization* [12, 13]. For example, it would be very sensitive if a young migrant woman is asked if she engages in commercial sex; it would be less sensitive however if she is asked to rate young women she does not know or even in a different country. CLT provides a new conceptual framework for conceiving the assessment of sensitive questions for survey studies.

### A Construal-level mechanism for measurement reliability

One mechanism underpinning the desensitization process described above is that respondents are very likely to “edit” their answers to a sensitive or intrusive question for self-assessment in order to maximize benefits, to conform with social norms, or to avoid threat of disclosure [1, 11]. In this case, the interpretation of the survey question by a respondent will be operated at the low, concrete, and context-related construal levels; the responses are thus less reliable because they are more likely to be purposefully edited, reducing information for reliable assessment of the target construct.

CLT-based research has demonstrated that social distance can function as a manipulation of construal level [14]. As the social distance between a respondent and the person or persons to be assessed increases, the interpretation of a survey question and the composition of the response will move to higher construal levels. As such, the response to the same question for socially distant others would be more reliable because the response will be (a) composited according to the established attitude or belief of the respondent [12, 14], (b) more abstractive, and (c) less likely to be affected by contextual factors [4, 12].

**Fig 1:**
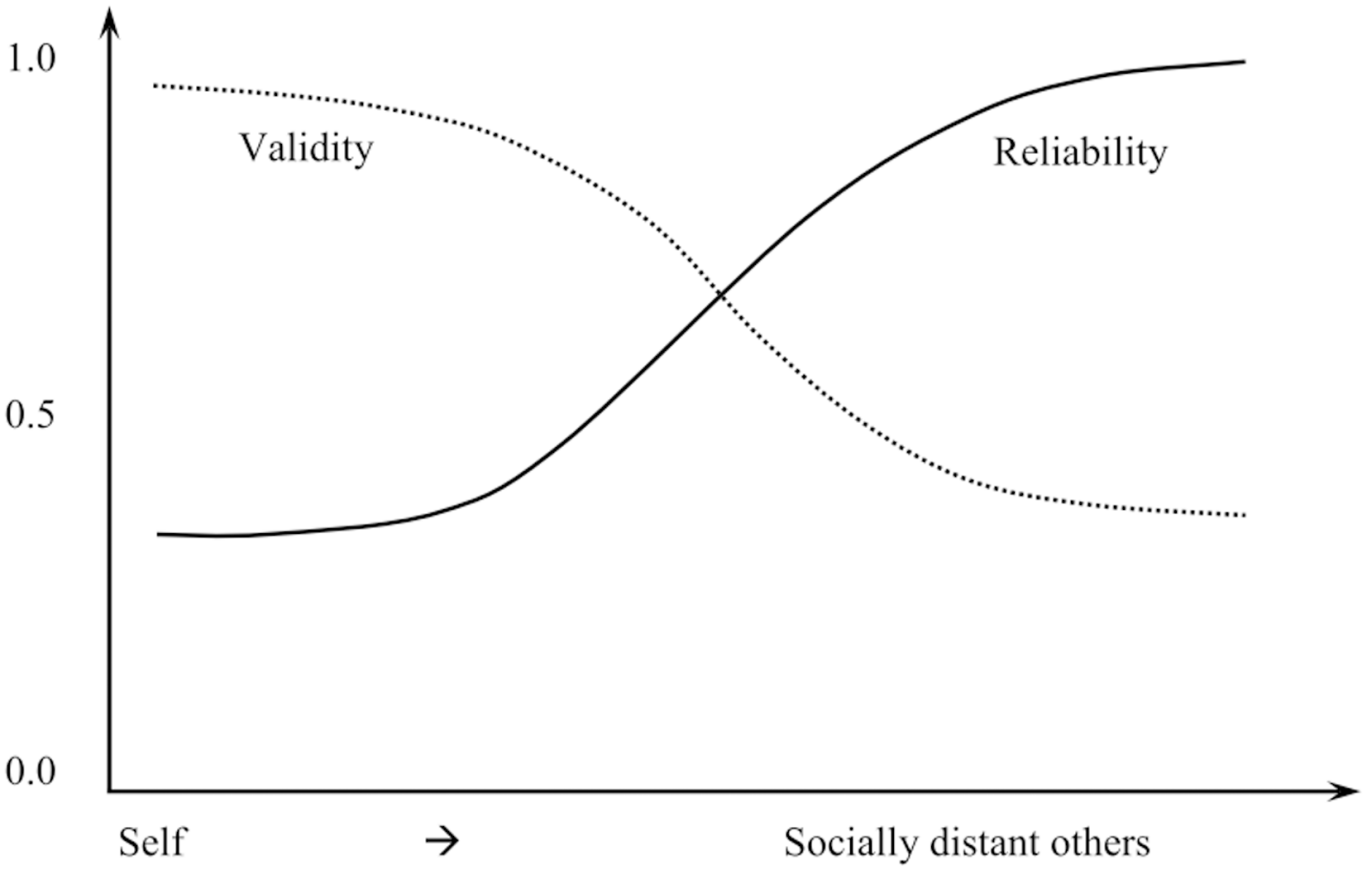
Hypothetical relationship between social distance and measurement reliability and validity.

From a measurement perspective, responses to a sensitive question contains two components, an *information component* reflecting the true answer to the question and an *artificial component (error)* due self-editing according to the context to avoid disclosure threat and to seek disclosure rewards. When answering a question for self-assessment or assessments of socially close others (e.g., family members, close friends), the artificial or non-informational component dominates, manifested as reductions in measurement reliability; while assessing socially distant others, the informational component dominates, manifested as increases in reliability. If this CLT-based hypothesis is applicable, the measurement reliability of a sensitive question would vary proportionately with social distance between the respondent and the assessed person(s) (the solid line in Fig 1).

### Maximize validity and reliability

Even if the reliability of a tool to assess sensitive questions may increase if respondents were asked to evaluate socially distant others, the validity of their responses may decline (the dashed line in Fig 1). It has been well established in sociology and social psychology that individuals with longer social distances differ more in attitudes and beliefs than individuals with shorter social distances [15–17]. Therefore, relatively to assessing individuals within a shorter social distance (i.e., family members and close friends), responses to a survey question for assessing individuals at longer social distances (i.e., acquaintances, strangers, and foreigners) contain less information for self-assessment.

The goal of a survey is to elicit not only consistent with high reliability but also correct and valid information regarding sensitive questions for the participants themselves [2]. We attempted to remedy this issued by consulting both the ancient philosophy of “judging others with one’s own beliefs” [18] and the modern social psychological theory of “false-consensus effect” [19]. According to these thoughts and previous discussion, an individual’s assessment of the attitudes and beliefs of others toward a sensitive question also contains a certain amount of information about his or her own attitudes and beliefs, although the amount of information may decline with social distance. Therefore a combination of self-assessment with the assessments of socially distant others may provide more valid data than using only self-assessment.

### CLT-based measurement techniques

According to the psychometric principles of latent variables and measurement modeling [20], we can hypothesize that responses to a sensitive question provided by a respondent for oneself and for socially distant others are all determined by one latent construct: *a respondent’s “real” attitude*, *or belief*. Therefore, it is possible to develop a technique for assessing sensitive questions with both high reliability and high validity by asking respondents the same questions for self-assessment and assessment of several groups of socially distant others. With data collected for self-assessment and the assessment of socially distant others, we can also separate the true attitudes and beliefs of the participants as a common factor from their perspectives on socially distant others using the analytical methods for multi-rater multi-traits, including the bifactor [21–23] and the trifactor [24] modeling analysis method. We termed this method *CLT-based measurement technique*.

## Methods

### Ethics statement

The survey protocol and the survey questionnaire were approved by the corresponding Institutional Review Boards (IRB) at Wuhan Center for Disease Prevention and Control, Wayne State University, and the University of Florida. Written consent was obtained from all participants before the survey.

### Participants and sampling

Participants were adult rural-to-urban migrants recruited in Wuhan, China. As the capital of Hubei Province, Wuhan is located in central China and has a per capita GDP of $10,335 and a total urban population of 10 million, of which 1.5 million are rural-to-urban migrants (Stastical Bureau of Wuhan, 2012). We elected to use data collected from a Chinese sample because Chinese are known as a conservative study population with regard to sex and sexual risk behavior [25]; China also experiences rapid changes in sexual attitudes, beliefs and behavior with increased exposure to the more sexually liberal western culture in recent decades [25, 26]. The increased diversity in attitudes, beliefs and behaviors related to sex makes the Chinese a suitable population for testing the new approach. The study sample was selected using a multi-stage random sampling strategy.

Data were collected during 2011–2013 using the Migrant Health and Behavior Questionnaire [27], administered using Audio Computer-Assisted Self-Interview. The interview was conducted in a private room in the participant’s home or another place of the participant’s preference (often a nearby community health center). Among the 1,293 participants who finished the survey, 161 were excluded as they indicated in the survey that less than 80% of their answers to the survey were reliable, yielding a final sample of 1,132.

De-identified survey data used for this study are available as Supporting Information (S1 Data).

### The Brief Sexual Openness Scale (BSOS)

The BSOS contains five items assessing attitudes toward five specific sexual behaviors: (1) premarital sex, (2) multiple sexual partners, (3) homosexuality, (4) extramarital sex, and (5) commercial sex. This scale was previously developed through a pilot study (see Appendix). An evaluation analysis of the scale with a pilot sample of rural-to-urban migrants (n = 77, mean age = 24.3) showed excellent reliability (alpha = .90).

### The novel CLT-based BSOS tool

The devised CLT-based BSOS consists of four components: (1) Self-assessment (as the origin), and assessment of (2) rural residents (shorter distance), (3) urban residents (longer distance), and (4) foreigners (longest distance). For self-assessment, participants were asked the degree to which they themselves agree with each of the five sexual behaviors using a 5-point Likert scale with 1 (*strongly disagree*) to 5 (*strongly agree*). For assessing each of the other three socially distant groups, participants were asked to estimate how many people in the groups may agree with the five statements, with answer options of 1 (*none or few*), 2 (*less than a half*), 3 (*about a half*), 4 (*more than a half*), and 5 (*almost all*). Data for the four social groups with a total of 20 items were included for psychometric analysis.

The CLT-based multi-component measurement technique was established and previously tested with pilot data from the same rural-to-urban migrant sample (n = 77) that were used to establish the BSOS as described in the previous section. Results from the pilot analysis indicated that the Cronbach alpha of BSOS varied from .90 for self-assessment to .95 for assessing urban residents, further to .96 for assessing foreigners. The Cronbach alpha of the BSOS as CTL-based tool was .94. The results (S1 Text) and de-identified survey data (S2 Data) of the pilot study are available as Supporting Information.

### Variables for assessing predictive validity

#### Sexual desire

Sexual desire was measured using the Simple Sexual Desire Scale we devised. It contains four items asking the respondents: “How often do you: (1) Think about sex during your free time? (2) Have strong feelings of sexual desire? (3) Talk about sex with others? (4) Notice sexually attractive persons around?” Response options were: 1 (*never)*, 2 (*occasionally)*, 3 (*not every day*, *at least once a week)*, 4 (*one or two times per day)*, 5 (*several times per day)*. The scale was pilot tested with adequate reliability (α = .91) and the mean score was used with a higher score indicating stronger desire for sex.

#### Dating frequency

This variable was assessed using one question: “How often have you had a date with someone in the past three months?” The answer options were 1 = “almost every day”, 2 = “multiple times in a week”, 3 = “about once a week”, 4 = “2–3 times a month”, 5 = “about once a month”, 6 = “occasional”, and 7 = “none”. The responses were reversely coded for analysis such that larger numbers indicating higher dating frequencies.

#### Age of first sex

For all participants who responded positively to the question, “Please recall from the time when you can recall till today. During this period, have you ever had sex with anyone, including with the same gender and any sexual behavior through vagina, anus or mouth?” they were further asked, “How old were you when you had sex the first time in life?” Reported age in years was used in analysis.

#### Number of sex partners in lifetime

The number of lifetime sex partners was assessed based on participants’ responses to the question, “Up to now, with how many persons have you had sexual intercourse, including your spouse, lovers, and strangers?” A zero was assigned to those who did not have sex so that these subjects were included in the analysis.

#### History of sexually transmitted diseases

History of sexually transmitted diseases (STD) was measured through self-report. All participants were asked “Have you ever had sexually transmitted diseases?” For those who responded positively, their answers were confirmed with two follow-up questions: (1) “Please indicate the type of sexually transmitted diseases” with a list of commonly reported STD as answer options; (2) “How did you know that you had the disease?” (Answer options: doctor’s diagnosis, self-assessment, told by others, and other methods).

### Psychometric evaluation

Item responses were assessed using mean, standard deviation, median, and interquartile range. Cronbach’s alpha was computed for the BSOS as a *conventional measure* for four groups (self, rural resident, urban resident, and foreigner). As a CLT-based measure, the reliability of the BSOS was assessed using the latent variable model [28, 29]. Theoretical analysis and empirical data indicate that when a scale with multi-level structures, the reliability of this scale will be underestimated using the conventional Cronbach alpha coefficient [28]. In assessing the reliability of the scale, the following criteria were used: .70 ≤ α < .80 (acceptable), .80 ≤ α< .90 (very good), and ≥ .90 (excellent).

To assess the structural validity of the CLT-based scale, we first conducted an exploratory factor analysis (EFA) to test the potential of a second-order one-factor model, assuming the existence of one factor for sexual openness with the four assessments (i.e., migrants themselves, rural residents, urban residents, and foreigners) as the first-order subfactors, which were further assumed to be determined by the more general second-order latent factor “sexual openness”. We further validate the one-factor model through confirmatory factor analysis (CFA). The data-model fit was assessed with the following: Comparative Fit Index (CFI) > .90, Tucker-Lewis Index (TLI) > .90, and root mean square error of approximation (RMSEA) < .05[30].

Following the one-factor modeling analysis, a tri-factor model was constructed and used to extract the common factor measuring sexual openness from the four perspective factor (i.e., the migrants themselves, and their perspectives of the rural residents, the urban residents and the foreigners), and the five item-level factors. The trifactor model was constructed based on the method devised for analyzing multi-raters and multi-traits data [24, 31], migrants’ self-assessment and their assessment of socially distant others were used in the place where different raters (informants) were modeled. The same data-model fit criteria for the one-factor CFA above were also used for assessing the trifactor model.

Predictive validity of the BSOS was assessed at three levels. First, four regression models were constructed to predict the conventional computed BSOS scores for migrants and three socially distant groups in predicting the five outcome variables. Second, two regression models were used to assess the one-factor BSOS scale in predicting the outcome variables, one for the sum score and another for the latent factor score. Lastly, five trifactor models were constructed to predict the five outcome variables. With this approach, the predictive validity of the common factor was adjusted for both perspective and item-specific factors while the relationship between migrants’ self-perspective and an outcome variable provides a measure of shared bias between self-assessment in BSOS and the outcome. In conducting the validity analysis, age, gender, and marital status were included as covariates in the model.

The reliability and validity of the various CLT-based BSOS were compared with those of the conventional BSOS to evaluate the superiority of the CLT-based approach in assessing sensitive questions. For all psychometric analyses, the type I error was set at p < .05 level (two-sided). The one-factor and the trifactor modeling analyses were conducted using the software AMOS 22.0 (IBM Corp., Armonk, NY) while data processing and other statistical analyses were completed using the commercial software SAS version 9.3 (SAS Institute Inc, Cary, NC).

## Results

### Sample characteristics

The study sample consisted of 1,132 participants with the mean age of 32.5 (SD = 7.9) years; 49.6% were female and 78.4% currently married. Approximately two-thirds had a middle school or lower level of education (67.3%), and 64.8% migrated to the city over 5 years ago. The monthly income was between 160 and 320 US dollars.

### Item response and reliability

Results in Table 1 show that the mean scores of individual items varied from a minimum of 1.52 (*SD* = 0.93) for self-assessment of the attitudes toward “commercial sex” to 2.84 (*SD* = 1.45) for assessing foreigners regarding the attitudes toward “premarital sex”.

**Table 1:** Item responses and reliability of the Brief Sexual Openness Scale (BSOS)–Conventional and CLT-based.

Scale item	Mean (SD)	Median (IQR)	Item-total correlation	
			Conventional	CLT- Based
**Sexual openness**	**2.08(0.76)**	**2.00(1.45–2.60)**		**α = 0.96**
***Assessment of self***	**1.76(0.79)**	**1.60(1.00–2.20)**	**α = 0.81**	
1. premarital sex	2.31(1.22)	2.00(1.00–3.00)	.80	.34
2. multiple sexual partners	1.64(1.03)	1.00(1.00–2.00)	.58	.19
3. homosexuality	1.79(1.09)	1.00(1.00–2.00)	.79	.24
4. extramarital sex	1.56(0.97)	1.00(1.00–2.00)	.74	.30
5. commercial sex	1.52(0.93)	1.00(1.00–2.00)	.75	.35
***Assessment of rural residents***	**1.70(0.93)**	**1.40(1.00–2.00)**	**α = 0.90**	
1. premarital sex	1.94(1.18)	2.00(1.00–2.00)	.69	.65
2. multiple sexual partners	1.81(1.29)	1.00(1.00–2.00)	.65	.53
3. homosexuality	1.59(1.04)	1.00(1.00–2.00)	.78	.60
4. extramarital sex	1.62(1.06)	1.00(1.00–2.00)	.83	.63
5. commercial sex	1.56(0.98)	1.00(1.00–2.00)	.79	.60
***Assessment of urban residents***	**2.15(1.06)**	**2.00(1.00–3.00)**	**α = 0.94**	
1. premarital sex	2.40(1.28)	2.00(1.00–4.00)	.81	.75
2. multiple sexual partners	2.23(1.22)	2.00(1.00–3.00)	.80	.73
3. homosexuality	2.00(1.10)	2.00(1.00–3.00)	.82	.77
4. extramarital sex	2.13(1.18)	2.00(1.00–3.00)	.89	.78
5. commercial sex	2.01(1.14)	2.00(1.00–3.00)	.86	.76
***Assessment of foreigners***	**2.68(1.31)**	**2.80(1.20–4.00)**	**α = 0.97**	
1. premarital sex	2.84(1.45)	3.00(1.00–4.00)	.89	.67
2. multiple sexual partners	2.82(1.44)	3.00(1.00–4.00)	.88	.69
3. homosexuality	2.58(1.36)	2.00(1.00–4.00)	.91	.68
4. extramarital sex	2.65(1.38)	3.00(1.00–4.00)	.93	.70
5. commercial sex	2.57(1.37)	2.00(1.00–4.00)	.90	.70

CTL-based BSOS is based on all 20 items for the four groups as input while conventional BSOS only uses five items to assess each of the four groups.

Results in Table 1 indicate that consistent with our pilot result, when the BSOS was used as a conventional measure, Cronbach alpha coefficients from low to high were .81 for self-assessment, .90 for assessing rural residents, .94 for assessing urban residents, and .97 for assessing foreigners (Table 1). This increasing trend is consistent with our hypothesis of a positive relationship between measurement reliability and the social distance. More importantly, the reliability coefficient of the 20-item CLT-based BSOS estimated using the latent variable model was 0.96, substantially higher than 0.81 (18.5% increases), the reliability of the conventionally based BSOS.

### One-factor modeling analysis

Results from EFA indicated that the 20 items contained only four subfactors. Furthermore, the five items for migrants were significantly loaded *only* on one factor (eigenvalue = 1.14), so did the five items for rural residents (eigenvalue = 2.33), urban residents (eigenvalue = 2.82) and foreigners (eigenvalue = 8.62). This result provides preliminary data supporting the proposed second-order factor model for the CLT-based method.

**Fig 2:**
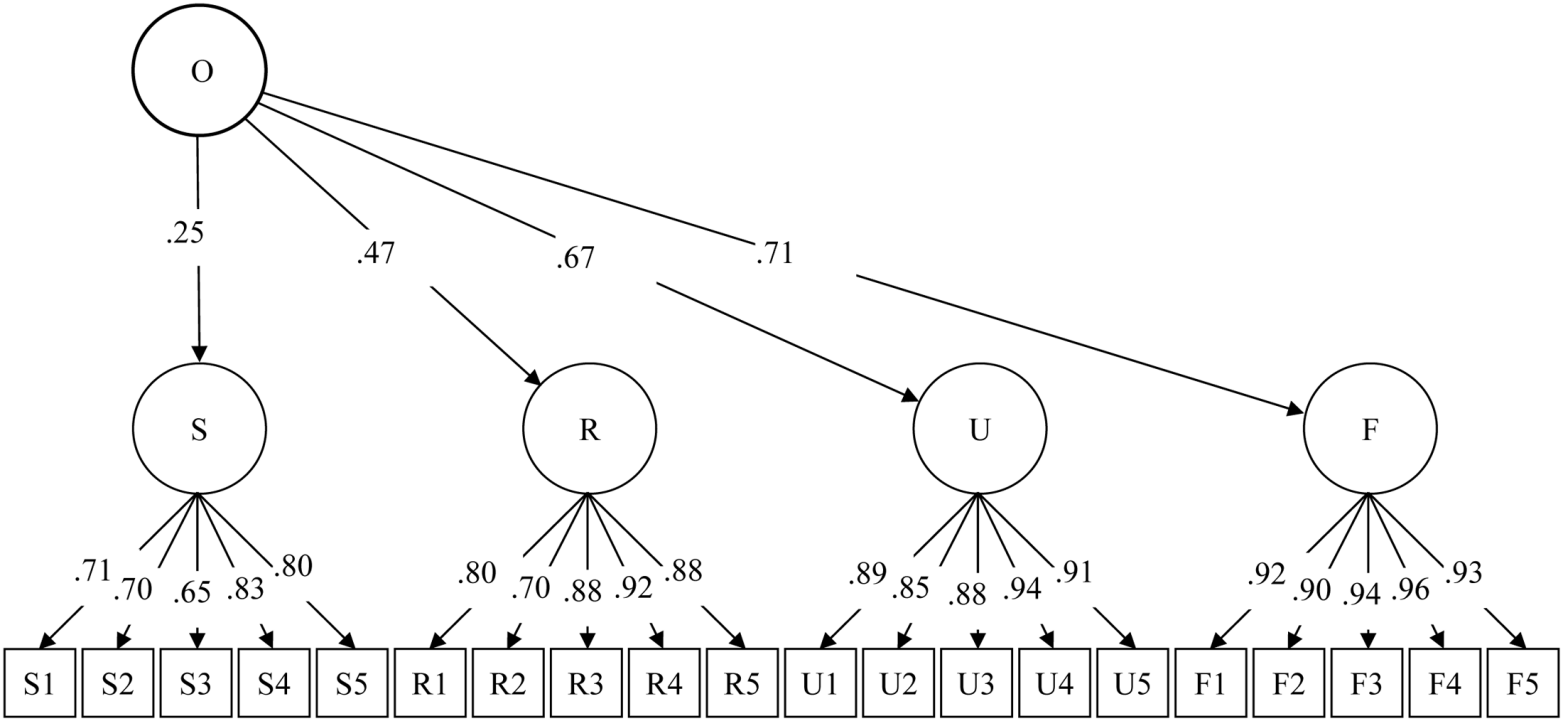
Measurement model of openness for rural-to-urban migrants sample. O = sexual openness. M = migrant self assessment. R = migrants’ assessment of rural residents. U = migrants’ assessment of urban residents. F = migrants’ assessment of foreigners. Data-model fit (without adding any covaiances among the measurement items): Chi-square = 1332, df = 168, CFI = .94, TLI = .93, RSMEA = .08. Two-group measurement modeling comparison indicated no gender differences.

Results from a one-factor CFA indicated a satisfactory data-model fit (CFI = .94, TLI = .93, RMSEA = .08), confirming the one-factor model for the CLT-based BSOS scale (Fig 2). Furthermore, results from the two-group comparative CFA [32, 33] indicated no gender differences in the measurement model, because change in CFI was a minor (ΔCFI = .002) when gender differences were considered. This result suggests that the CLT-based model is suitable for both males and females. Individual BSOS items all loaded properly on the four first-order factors (assessments of four groups), and the four first-order factors loaded on the second-order factor (sexual openness). The standardized structural loadings of the first-order factors from low to high were .25 for self-assessment, .47 for assessment of rural residents, .67 for assessment of urban residents, and .71 for assessment of foreigners (Fig 2).

### Trifactor modeling analysis

**Fig 3:**
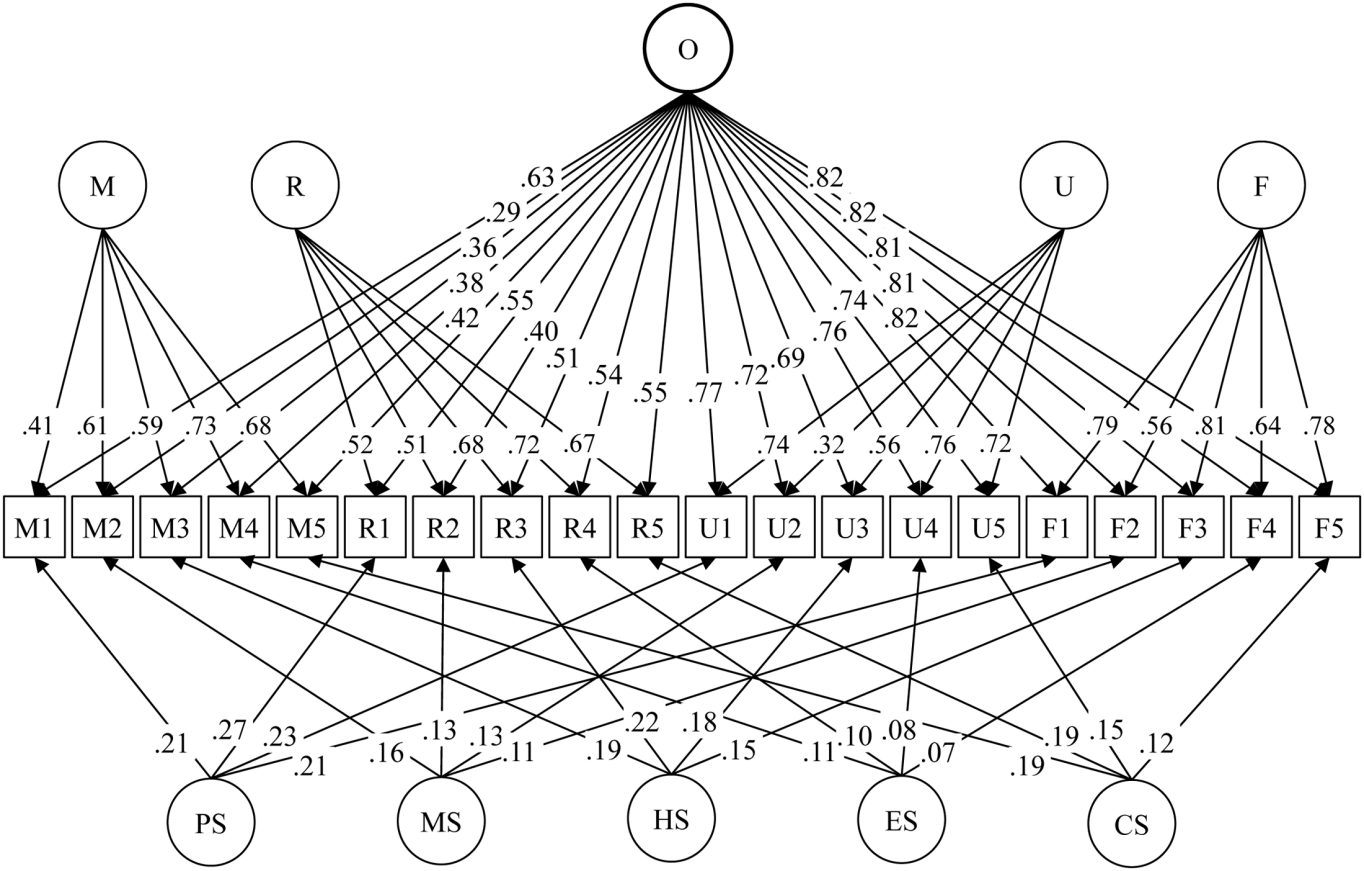
Trifactor modeling analysis of the CLT-based BSOS. O = the common factor for sexual openness. M = perspective factor of migrants themselves, R = migrants’ perspective of rural residents, U = migrants’ perspective of urban residents, and F = migrants’ perspective of foreigners. PS = Subfator for the four items related to premarital sex, MS = subfator for the four items related to mutliple sex partners, HS = subfator for the four items related to homosexuality, ES = subfator for the four items related to extramarital sex, and CS = subfators for the four items related to commercial sex. Data-model fit (without adding any covaiances among the measurement items): Chi-square = 1218, df = 161, CFI = .95, TLI = .94, RSMEA = .08. Two-group comparison indicated no significant gender differences in the trifactor CLT-based BSOS model.

Fig 3 presents the constructed trifactor model. The factor loading for all items across the four assessments were set to be equal as constraints to solve the model, since the four assessments were made by one person [24]. Results in Fig 3 show satisfactory data-model fit (CFI = .95, TLI = .94, and RMSEA = .08). A common factor O (sexually openness) was successfully extracted with factor loadings substantially greater than those of the five specific subfactors and greater than many of those for the two perspective subfactors U (urban residents) and F (foreigners).

In addition to the common factor O, the perspective factor M provide a measure of the bias portion from migrants’ self assessment after the “true” portion was factored out to O. Other three perspective factors R, U and R represents migrants’ truse assessments of rural residents, urban residents and foreigners because the common part from migrants own pointviews were factored to the common factor O. The factor loadings were rather small for the five specific factors PS (premarital sex), MS (mutliple sex partners), HS (homosexuality), ES (extramarital sex), and CS (commercial sex), indicating significant and essential contributions of all items to the latent construct of sexual openniess measured with the CLT-based method.

**Fig 4:**
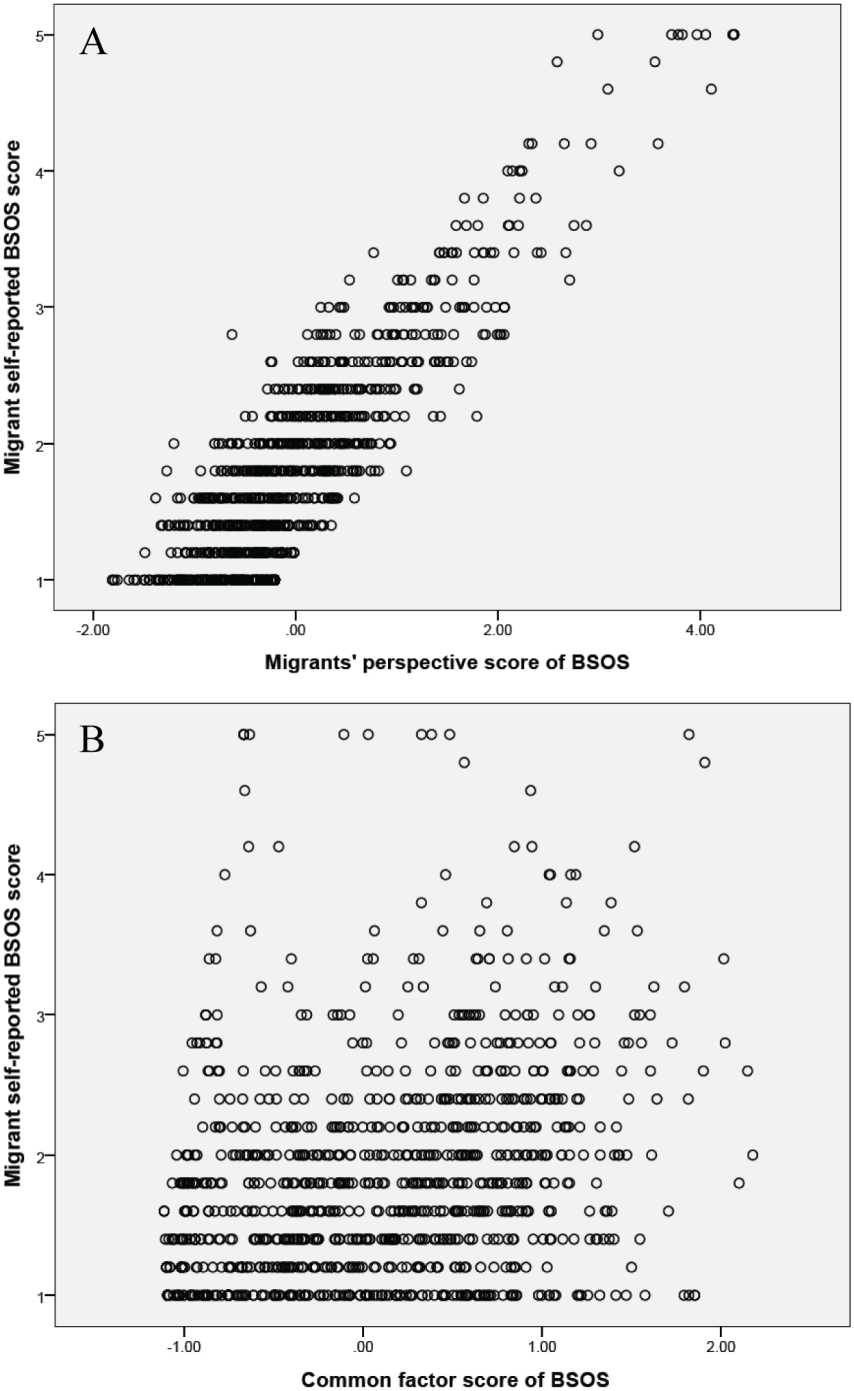
Plotting of the perspectiev factor M and the common factor O against BSOS scores directly summed up from migrants self-report. Latent scores for the common factor O (CLT-based sexual openness) and the perspective factor M (biased self-perspective of migrants) were derived from the trifactor CLT-based BSOS model (see Fig 3). For migrants’ perspective score of BSOS, mean (SD) = 0.00 (.90), median [interquartile range] = -.20, [-.54, .33). For common factor score of BSOS, mean (SD) = 0.00 (.77), median [interquartile range] = -.06 [-.68, .61).

Results in Fig 4A indicate that a very high correlation (*r* = .86, *p* < .001) between perspective factor M derived from the trifactor model and the migrants’ self-reported BSOS scores through the conventional method; while results in Fig 4B indicate a rather low correlation (*r* = .35, *p* < .001) between the common factror O derived from the trifactor model and the self-reported BSOS score. This result suggest that the M factor containes the biased information probably due to self-editing while the common factor O contains the corrected measurement of sexual openness.

### Predictive validity

Results from the various predictive validity analyses are presented in Table 2. First of all, the BSOS significantly predicted all five outcomes as a conventional measure, significantly predicted all but one (STD history) as a one-factor CLT-based measure, and predicted three of the five as a tri-factor CLT-based measure. Second, migrants as a perspective factor derived from the trifactor model that measures self-report bias significantly predicted all five outcomes with standard regression coefficients from 0.11 (p < .01) in predicting number of sexual partners to-.22 (p < .01) in predicting age of first sex. These results suggest that the correlations between the self-assessment as a conventional measure and the five outcome variables were all confounded by errors from self-report. The association was primarily attributed to a shared bias, or misreporting of both the predictor and the outcome variables. Third, a comparison of the result from the self-assessment with that of the common factor indicates that BSOS as a conventional measure significantly underestimated its association with sexual desire (coefficients: .22 vs. .44, Z = 2.00, p < .05), and overestimated the association with STD history (.11 vs .01, Z = 11.00, p < .01). Lastly, after the common factor was extracted from the data, migrants’ perspectives of the three socially distant groups were less likely to predict the outcome variables. These results suggest that it is primarily the common factor from migrants themselves made their assessments of socially distant others seemingly valid in predicting the outcomes.

**Table 2:** Predictive validity of the Brief Sexual Openness Scales (BSOS) as a conventional and a CLT-based measure (standardized regression coefficients), N = 1,132.

Measurement of seuxal openness	Sexual desire	Date frequency	Age of first sex	No. sexual partners	STD history
**Conventional measurement**					
**Self-assessment**	.22**	.14**	-.25**	.13**	.11**
**Assessment of others**					
Rural residents	.31**	.09**	-.12**	.10**	.09**
Urban residents	.35**	.05	-.18**	.10**	.04
Foreigners	.29**	.04	-.16**	.02	-.03
**CLT-based measurement**					
**One-factor model**					
Sum scale scores	.41**	.10**	-.24**	.12**	.05
Latent factor scores	.38**	.07*	-.20**	.08*	.02
**Trifactor model**					
Common factor	.44**	.08	-.31**	.16**	.01
Perspective factors					
*Migrants selves*	.15**	.15**	-.22**	.11**	.14**
*Rural residents*	.14**	.07	.02	.03	.09*
*Urban residents*	.09	.03	-.02	.03	.10
*Foreigners*	.05	.05	-.01	-.11	.01

Regression models were used to assess the conventional and one-factor-based BSOS, and structural equation models were used to assess trifactor-based BSOS. Age, gender, and marital status were entered as controls for all analyses. Analysis of age of first sexual intercourse was limited to participants who reported ever having had sex (n = 446).

*p < 0.05.

**p < 0.01.

## Discussion

Obtaining reliable and valid data on sensitive questions represents a longstanding challenge and an important task in modern survey research, particularly for HIV-related survey research [1]. In this study, we have demonstrated a novel CLT-based measurement technique to obtain reliable and valid data, using the Brief Sexual Openness Scale as an example. Different from many traditional methods/techniques that attempted to enhance reliability by circumventing the sensitivity of survey questions; our method is based on a direct measurement of sensitive questions. The method was pilot-tested previously, and we further validated it in this study with new data collected from a large random sample of Chinese rural-to-urban migrants. This subpopulation has increased exposure to modern sex culture along with the open policy in China [26]. China is a country known for its long history of conservative culture regarding sex [25] with increased exposure to the more liberal culture in the recent past [25, 26]. In addition to validating the proposed CLT-based technique for measuring sexual openness, these study findings also provide support for the hypotheses regarding the relationship between CLT and reliability and validity. To the best of our knowledge, this is the first study of its type to tackle sensitive questions for better survey data. Findings of our study suggest that the CLT-based technique could be a promising method to measure sensitive questions.

### Adequate reliability and validity of CLT-based measures

Consistent with our hypothesis, findings of this study clearly demonstrate the reliability and validity of CLT-based measurement of sensitive questions, in this case the attitude of sexual openness, for rural-to-urban migrants in China. The reliability of the BSOS was *excellent* (alpha = .96) if devised based on CLT but only *good* (alpha = .81) if used as a conventional tool. More exciting than the high reliability is the validity of the CLT-based measure in correctly predicting outcome measures after controlling for covariates. In addition to measuring sexual openness, the findings of this study suggest the potential for addressing other sensitive topics using the same technique for different populations in different cultural settings.

The extraction of the common factor and the perspective factors of the participants with the trifactor modeling analysis enable us to assess the shared bias in both the predictor and the outcome variables. After controlling for the shared bias, the adjusted association between the CLT-based measures and the outcomes provides a more accurate assessment of the validity of the instrument. In addition to assess validity, the trifactor-based approach can also assist researchers to assess the quality of variables as outcome measures. For example, self-reported STD history and self-reported dating frequency are not good outcome measures to validate BSOS. These two variables were significantly associated with migrants’ perspective of sexual openness but not significantly associated with the common factor of sexual openness.

### Construal levels and measurement reliability

The high reliability of the CLT-based measurement was supported by the evidence that there was a positive relationship between the reliability (as measured by the Cronbach’s alpha) and social distance (e.g., from the rural-to-urban migrants themselves, to rural residents, urban residents, and foreigners). This finding provides quantitative data supporting the utility of CLT in assessing sensitive questions. When people assess themselves, the cognitive process occurs at a low construal level, and is more context-oriented, and thus may situationally deviate from their inner viewpoints, values, and beliefs and contain more errors and less information for assessment. When people assess others, the same process occurs at a higher construal level without adequate information, and is less context-dependent, and more likely to reflect the stable inner latent viewpoints, attitudes, and beliefs [14], and therefore contain more reliable information for measuring the true attitudes and beliefs of an individual.

When reporting on one’s assessments of others, people gradually “disarm” themselves from the “self-censorship” as social distance increases. Along with the increase in social distance, sensitive questions now becomes less sensitive and a socially desirable answer becomes less desirable; therefore the responses to a sensitive question contain more information, and less error, becoming more reliable for measurement. Although various empirical studies have examined construal level by dichotomizing it into high and low [14, 34], this study is the first to support CLT with Cronbach alpha coefficients, a continuous and quantitative measure.

### Measures at various construal levels and validity

Consistent also with our hypothesis, the high validity of a CLT-based measure is supported by the data obtained from respondents’ assessments of various others with different social distances. Guided by the ancient philosophy of “judging others with one’s own beliefs” [18] and the *false-consensus effect* of modern social psychology [19], we believe that although survey respondents may process their answers to sensitive questions at different construal levels for people at different social distances, the survey responses are formed from the same latent viewpoints, attitudes, and beliefs. This hypothesis was directly supported by results from confirmatory factor analysis using both one-factor and trifactor models. In addition to self-assessment, the assessments of three other groups (rural residents, urban residents, and foreigners) regarding their sexual openness all contributed to the latent construct of “sexual openness” of the survey respondents as implicitly indicated by the results from the one-factor modeling analysis and further provided explicitly by the common factor extracted through trifactor modeling analysis.

Lastly, the relationship between a CLT-based BSOS and an outcome variable was stronger for less sensitive variables (e.g., sexual desire and age of first sex) than for more sensitive ones (e.g., multiple sexual partners and STD histories) when the common factor was used as predictor. The distinguishing sensitivity levels of these questions have been documented in published studies [26]. The CLT-based BSOS measures were better predictors for the less sensitive behavioral outcomes, whereas the conventional BSOS measures were strong predictors of the more sensitive outcomes. Findings from our analysis indicate that this phenomenon is due to a shared or common source of bias for both the predictor and outcome variables [35]. The conventionally measured BSOS was associated with more sensitive outcome measures (e.g. frequency of dating and STD history) because both were misreported in the same direction. Even if two variables are not associated, this shared underreporting will lead to a significant association as we show in our analysis. When the BSOS is used as a conventional self-report measure, sexual openness and the outcome variables are all subjective to sensitivity-related bias. Therefore, a portion of the association between the predictor and outcome variables is due to the “shared” bias between them. This finding further supports superiority of the CLT-based BSOS as a valid tool over the conventional BSOS in measuring sensitive questions.

### Limitations and conclusion

There are limitations to this study. First, we tested the CLT-based technique with only one sensitive construct, sexual openness, although five questions are asked. Additional studies with more questions of varying sensitivity levels are needed. Second, sensitivity of a survey questions may vary by cultural and other demographic factors (e.g., gender) [8]. We only tested the CLT-based method among young adults from China. Although the Chinese may become more diverse with regarding to sexual attitudes and beliefs after increased exposure to the western culture in the past several decades, making this research findings more generalizable to different settings, additional studies are required to assess the performance of this approach among people in across different countries and places across the globe. Given these limitations, findings of this study highly suggest that CTL-based measurement technique could be a promising approach to assess sensitive questions in general as well as to meet the urgent need for HIV-related survey studies.

## Supporting Information

S1 Data Dataset for the main study. (CSV)

S2 Data Dataset for the pilot study. (CSV)

S1 Text Summary of results for pilot study. (DOCX)

## References

[1] Tourangeau R, Yan T. Sensitive questions in surveys. Psychological Bulletin. 2007;133(5):859–83. 2007-12463-007. 17723033 10.1037/0033-2909.133.5.859

[2] Krumpal I. Determinants of social desirability bias in sensitive surveys: a literature review. Qual Quant. 2013;47(4):2025–47. 10.1007/s11135-011-9640-9 ISI:000316267500014.

[3] Yip PSF. Sex knowledge, attitudes, and high-risk sexual behaviors among unmarried youth in Hong Kong. BMC public health. 2013;13(1):691 10.1186/1471-2458-13-691 23895326 PMC3729422

[4] Fisher RJ. Social Desirability Bias and the Validity of Indirect Questioning. J Consum Res. 1993;20(2):303–15. ISI:A1993LY49000010.

[5] Warner SL. Randomized-Response—a Survey Technique for Eliminating Evasive Answer Bias. J Am Stat Assoc. 1965;60(309):63–9. ISI:A1965CKX1300005. 12261830

[6] Sirken MG. Household Surveys with Multiplicity. J Am Stat Assoc. 1970;65(329):257–66. 10.2307/2283590

[7] Roese NJ, Jamieson DW. 20 Years of Bogus Pipeline Research—a Critical-Review and Metaanalysis. Psychological Bulletin. 1993;114(2):363–75. ISI:A1993LX35200009.

[8] Albaum G, Roster CA, Smith SM. A Cross National Study of Topic Sensitivity: Implications for Web-Based Surveys. Journal of Marketing Development and Competitiveness. 2012;6(5):71–82.

[9] van Houwelingen G. Something to rely on: The influence of stable and fleeting drivers on moral behavior 2015.

[10] Trope Y, Liberman N. Temporal construal. Psychol Rev. 2003;110(3):403–21. ISI:000184112000001. 12885109 10.1037/0033-295x.110.3.403

[11] Trope Y, Liberman N. Construal-level theory of psychological distance. Psychol Rev. 2010;117(2):440–63. Epub 2010/05/05. 10.1037/a0018963 20438233 PMC3152826

[12] Wright SA. Using construal level theory to deter the social desirability bias. 2013.

[13] Kim M-S. Non-western perspectives on human communication: Implications for theory and practice. Thousand Oaks, CA: Sage Publications, Inc.; 2002.

[14] Liberman N, Trope Y, Stephan E. Psychological distance In: Kruglanski AW, Higgins ET, editors. Social Psychology: Handbook of Basic Principles. New York Guilford Press; 2007.

[15] Smith JA, McPherson M, Smith-Lovin L. Social Distance in the United States: Sex, Race, Religion, Age, and Education Homophily among Confidants, 1985 to 2004. Am Sociol Rev. 2014;79(3):432–56. ISI:000340424400004.

[16] Holtz R. Group cohesion, attitude projection, and opinion certainty: Beyond interaction. Group Dyn-Theor Res. 2004;8(2):112–25. ISI:000222023700003.

[17] Liviatan I, Trope Y, Liberman N. Interpersonal similarity as a social distance dimension: Implications for perception of others' actions. Journal of experimental social psychology. 2008;44(5):1256–69. ISI:000259162800005. 19352440 10.1016/j.jesp.2008.04.007 PMC2665912

[18] Han Y. Commentary on Han Poetry. China: unknown 150 B.C.

[19] Ross L, Greene D, House P. The “false consensus effect”: An egocentric bias in social perception and attribution processes. Journal of experimental social psychology. 1977;13(3):279–301.

[20] Borsboom D. The theoretical status of latent variables. Psychol Rev. 2003;110(2):203–19. 12747522 10.1037/0033-295X.110.2.203

[21] Holzinger KJ, Swineford F. The Bi-Factor Method. Psychometrika. 1937;2(1):41–54. 10.1007/Bf02287965 ISI:000203818600005.

[22] Cai L, Yang JS, Hansen M. Generalized Full-Information Item Bifactor Analysis. Psychol Methods. 2011;16(3):221–48. 10.1037/A0023350 ISI:000294927800001. 21534682 PMC3150629

[23] Gibbons RD, Bock RD, Hedeker D, Weiss DJ, Segawa E, Bhaumik DK, et al Full-information item bifactor analysis of graded response data. Appl Psych Meas. 2007;31(1):4–19. 10.1177/0146621606289485 ISI:000242723200001.

[24] Bauer DJ, Howard AL, Baldasaro RE, Curran PJ, Hussong AM, Chassin L, et al A Trifactor Model for Integrating Ratings Across Multiple Informants. Psychol Methods. 2013;18(4):475–93. 10.1037/A0032475 ISI:000329996000003. 24079932 PMC3964937

[25] Zheng WJ, Zhou XD, Wang XL, Hesketh T. Sociosexuality in Mainland China. Archives of sexual behavior. 2013:1–9. 23740470 10.1007/s10508-013-0097-x

[26] Zheng W, Zhou X, Zhou C, Liu W, Li L, Hesketh T. Detraditionalisation and attitudes to sex outside marriage in China. Culture, health & sexuality. 2011;13(05):497–511. 10.1080/13691058.2011.563866 21452090

[27] Chen X, Stanton B, Gong J, Fang X, Li X. Personal Social Capital Scale: an instrument for health and behavioral research. Health Educ Res. 2009;24(2):306–17. Epub 2008/05/13. cyn020 [pii] 10.1093/her/cyn020 . 18469318

[28] Raykov T, Shrout PE. Reliability of scales with general structure: Point and interval estimation using a structural equation modeling approach. Structural Equation Modeling. 2002;9(2):195–212. 10.1207/S15328007sem0902_3 ISI:000180828700003.

[29] Raykov T, Marcoulides GA. Evaluation of Validity and Reliability for Hierarchical Scales Using Latent Variable Modeling. Struct Equ Modeling. 2012;19(3):495–508. 10.1080/10705511.2012.687675 ISI:000306997100009.

[30] Kline RB. Principles and practice of structural equation modeling. New York: Guilford Press; 2011.

[31] Campbell DT, Fiske DW. Convergent and discriminant validation by the multitrait-multimethod matrix. Psychol Bull. 1959;56(2):81–105. Epub 1959/03/01. . 13634291

[32] Byrne BM. Testing for multigroup invariance using AMOS graphics: A road less traveled. Struct Equ Modeling. 2004;11(2):272–300. 10.1207/s15328007sem1102_8 ISI:000221805000008.

[33] Byrne BM. Structural Equation Modeling With AMOS: Basic Concepts, Applications, and Programming. New York, NY: Routledge; 2009.

[34] Fujita K, Carnevale JJ. Transcending temptation through abstraction: The role of construal level in self-control. Current directions in psychological science: a journal of the American Psychological Society. 2012;21(4):248–52.

[35] Spector PE. Method variance in organizational research—Truth or urban legend? Organ Res Methods. 2006;9(2):221–32. ISI:000237110500005.

