# Supplementary material for: Development and Evaluation of the Brief Sexual Openness Scale—A Construal Level Theory Based Approach
Source: PLoS One. 2015 Aug 26;10(8):e0136683. doi: 10.1371/journal.pone.0136683 (PMC4550254; doi:10.1371/journal.pone.0136683)
Supplement: S1 Text — (DOCX) [file pone.0136683.s003.docx]

**Appendix 1:** **Pilot Study of the Brief Sexual Openness Scale (BSOS) and an Exploration of a CLT-based Measure of BSOS**

**1. Purpose**

It is a long standing challenge to assess sensitive questions in HIV survey studies [[1](#_ENREF_1)]. The purposes of this study are two folds (1) to establish the Brief Sexual Openness Scale (BSOS), including the assessment of item response and reliability and (2) to explore the potentials to devise a construal level theory (CLT)-based measurement approach for better assessing sensitive questions (using BSOS as an example) in survey studies to improve HIV research.

**2 Methods**

**2.1. Participants and sampling**

Participants were rural-to-urban migrants from Wuhan, the capital city of Hubei

Province, China. More details about the sampling procedure and sample characteristics were reported elsewhere [[2](#_ENREF_2)]. Briefly, the participants were sampled and recruited at Wuhan Centers for Disease Prevention and Control (CDC) where rural migrants are instructed to conduct annual physical check-ups as part of their employment requirements in the city. Recruitment was completed by trained investigators in the waiting room of the CDC clinic. Among the 86 rural migrants approached, 77 (90%) agreed to participate and completed the survey questionnaire after signing the consent form. Data collection was completed in 2011.

**2.2. Brief Sexual Openness Scale (BSOS)**

The Brief Sexual Openness Scale (BSOS) was developed based on literature review and previous HIV research to assess attitudes toward five sexual behaviors: (1) premarital sex, (2) multiple sexual partners, (3) homosexuality, (4) extramarital sex, and (5) commercial sex. Migrants’ self-assessment of these items was rated on a standard 5-point Likert scale from 1 (*strongly disagree*) to 5 (*strongly agree*).

In addition to self-assessment, the participants were asked to assess other two socially distant groups (i.e., urban residents in general and foreigners) regarding the five sexual behaviors using the question: “In your opinion, please estimate how many of [urban residents or foreigners] also agree with the five sex-related behaviors?” The questions were assessed with another 5-point scale ranging from 1 (*none or few*) to 5 (*almost all*).

**2.3. Contrual-Level Theory Based BSOS**

According to CLT, data from migrants’ self-assessment are less reliable than their assessment of socially distant groups [[3](#_ENREF_3),[4](#_ENREF_4)]. We thus believe that a combination of data from migrants’ self-assessment with their assessments of the others will provide a more reliable measure then the conventional self-assessment. We then devised and tested the CLT-based assessment method by using the data for the three groups with a total of 15 items, and we termed this as CLT-based BSOS.

**2.4 Data processing and statistical analysis**

Data processing was completed using the software SAS, version 9.2 (SAS Institute, Cary, NC). Psychometric assessments were completed using AMOS 22.0 (IBM Corp., Armonk, NY).

**3. Results**

**3.1 Sample characteristics**

Among the 77 participants, 26 (33.8%) were male and 58 (75.3%) were unmarried. The mean age of the sample was 24.3 (SD = 5.8). Fifty-seven (74.0%) had middle or high school education and 75% had a monthly income lower than 160 US dollars.

**3.2 Item response and reliability**

Results in Table 1 indicate that the mean scores (SD) of individual items ranged from a minimum of 1.77 (1.16) for self-assessment of the attitudes toward “commercial sex” to a maximum of 3.40 (1.49) for their assessment of foreigners’ attitudes toward “premarital sex”. Cronbach’s alpha coefficients from low to high were .90 for self-assessment, .95 for assessment of urban residents, and .96 for assessment of foreigners. The alpha coefficient of CLT-based BSOS based on 15 items was .94.

**Table 1. Item responses and reliability of the Brief Sexual Openness Scale (BSOS) – Conventional and CLT-based**

| Scale item | Mean (SD) | Median (IQR) | Item-total correlation | |
| --- | --- | --- | --- | --- |
|  |  |  | Conventional | CLT-based |
| **Sexual openness** | **2.38(0.87)** | **2.40(1.80-3.00)** |  | **α =** **0.94** |
| ***Assessment of self*** | **2.12(1.08)** | **1.80(1.20-3.00)** | **α =** **0.90** |  |
| 1. premarital sex | 2.74(1.37) | 3.00(1.00-4.00) | .55 | .55 |
| 2. multiple sexual partners | 2.06(1.28) | 2.00(1.00-3.00) | .83 | .58 |
| 3. homosexuality | 2.22(1.33) | 2.00(1.00-3.00) | .76 | .56 |
| 4. extramarital sex | 1.82(1.22) | 1.00(1.00-2.00) | .84 | .53 |
| 5. commercial sex | 1.77(1.16) | 1.00(1.00-2.00) | .83 | .55 |
| ***Assessment of urban residents*** | **2.57(1.13)** | **2.60(1.60-3.40)** | **α=0.95** |  |
| 1. premarital sex | 2.95(1.32) | 3.00(2.00-4.00) | .83 | .79 |
| 2. multiple sexual partners | 2.75(1.33) | 3.00(1.00-4.00) | .88 | .81 |
| 3. homosexuality | 2.34(1.16) | 2.00(1.00-3.00) | .87 | .76 |
| 4. extramarital sex | 2.40(1.17) | 2.00(1.00-3.00) | .87 | .75 |
| 5. commercial sex | 2.41(1.19) | 2.00(1.00-3.00) | .87 | .74 |
| ***Assessment of foreigners*** | **3.07(1.30)** | **3.60(2.00-4.00)** | **α = 0.96** |  |
| 1. premarital sex | 3.40(1.49) | 4.00(2.00-5.00) | .84 | .74 |
| 2. multiple sexual partners | 3.35(1.43) | 4.00(2.00-4.00) | .86 | .80 |
| 3. homosexuality | 2.96(1.40) | 3.00(2.00-4.00) | .92 | .77 |
| 4. extramarital sex | 2.84(1.36) | 3.00(2.00-4.00) | .91 | .78 |
| 5. commercial sex | 2.80(1.39) | 3.00(1.00-4.00) | .85 | .73 |

**Note**: CTL-based BSOS is based on all 15 items for the three groups while conventional BSOS.

**3.3 Confirmatory factor analysis**

Results from the basic one-level CFA indicate a poor model-data fit (CFI = .86, TLI = .83, RMSEA = .16, χ2/df = 3.08). The data-model fit was improved by adding appropriate covariances according to the modification indices (CFI = .95, TLI = .94, RMSEA = .10, χ2/df = 1.75). A more complex second-order factor analysis was conducted, treating the three assessments (i.e., migrants themselves, urban residents, and foreigners) as three first-order factors, which were further assumed to be determined by the more general second-order latent factor “sexual openness”. Modeling results indicated a poor data-model (CFI = .86, TLI = .83, RMSEA = .16, χ2/df = 3.08) that was improved by adding appropriate covarnaces (CFI = .95, TLI = .94, RMSEA = .10, χ2/df = 1.75). Figure 1 presents the factor loadings for the second-order factor analysis.

S2

S3

S4

S5

S1

R2

R3

R4

R5

R1

U2

U3

U4

U5

U1

.42

.76

.99

.59

.87

.843

.89

.90

.83

.86

.92

.91

.92

.79

.82

.94

.98

.91

**Fig. 1. Measurement model of openness for rural-to-urban migrants sample**

*Note.* O = sexual openness. S = assessment of self. U = assessment of urban residents. F = assessment of foreigners. Model fit: CFI = .95, TLI = .94, RMSEA = .10, χ2/df = 1.75. Covariances were added following the modification indexes.

**4. Discussion and conclusions**

Results from our study indicated that BSOS, although short, has excellent reliability (alpha=.90) for assessing sexual openness among rural migrants in China. Results from CFAs support the potentials to establish a CLT-based BSOS. Furthermore, CLT-based BSOS appears to provide more reliable data than the conventional BSOS. Additional studies are needed to verify the CLT-based method.

References for pilot study

1. Tourangeau R, Yan T (2007) Sensitive questions in surveys. Psychological Bulletin 133: 859-883.

2. Chen X, Yu B, Gong J, Zeng J, MacDonell KK (2015) The Domestic Migration Stress Questionnaire (DMSQ): Development and psychometric assessment. Journal of Social Science Studies 2: 117-133.

3. Liberman N, Trope Y, Stephan E (2007) Psychological distance. In: Kruglanski AW, Higgins ET, editors. Social Psychology: Handbook of Basic Principles. New York Guilford Press.

4. Trope Y, Liberman N (2010) Construal-level theory of psychological distance. Psychological Review 117: 440-463.
